# Laxative‐induced contact burns from accidental ingestion of senna in a 2‐year‐old female

**DOI:** 10.1002/jpr3.70087

**Published:** 2025-09-28

**Authors:** Tolulope Olorunsogo, Christian O. Oarhe, Ahmad R. Miri

**Affiliations:** ^1^ Pediatric Gastroenterology, Hepatology and Nutrition University of Nebraska Medical Center, Children's Nebraska Omaha Nebraska USA; ^2^ Pediatric Emergency University of Nebraska Medical Center, Children's Nebraska Omaha Nebraska USA

**Keywords:** blisters, case report, pediatric, preventive measures, sennosides

## Abstract

Senna (sennosides) is a natural stimulant laxative containing anthraquinone glycosides, commonly used to treat constipation. We present the case of a healthy 2‐year‐old female (~12.5 kg) who is not yet toilet‐trained and accidentally ingested a single 15 mg chocolate‐flavored sennoside chew (1.2 mg/kg). This led to sharply demarcated erythema, pain, and blisters in her diaper area. In the emergency department, the blisters were treated by unroofing, topical mupirocin application, a petrolatum dressing, and a 5‐day course of oral cephalexin. The burn injury healed without complications at a follow‐up visit. This case highlights the risk of senna‐induced burns in diapered toddlers, as prolonged stool‐skin contact can cause blistering that mimics abusive injury. Inquiries about sennoside use should be made when perineal burns are observed in pre‐continent children to prevent misdiagnosis of child abuse. Preventive measures, including caregiver education, child‐resistant packaging, and safe medication storage, are essential.

## 1 INTRODUCTION

Sennosides contain anthraquinone glycosides that stimulate intestinal motility, making it a mainstay treatment for functional constipation in children.^1^ Although gastrointestinal cramping and diarrhea are recognized adverse effects, perineal contact dermatitis with blistering/occurrence of contact burn is rare, particularly in pediatric patients, but has been documented.^2^, ^3^, ^4^ Despite widespread availability and perceived safety, national poison control centers report that sennoside exposure remains a recurring toxicology concern in pediatrics, due to children's exploratory behavior and the attractive appearance of these substances.^5^ These injuries typically occur in pre‐continent toddlers after inadvertent ingestion and can be mistaken for scald or immersion burns, raising safety concerns.

## 2 CASE REPORT

**Figure 1:**
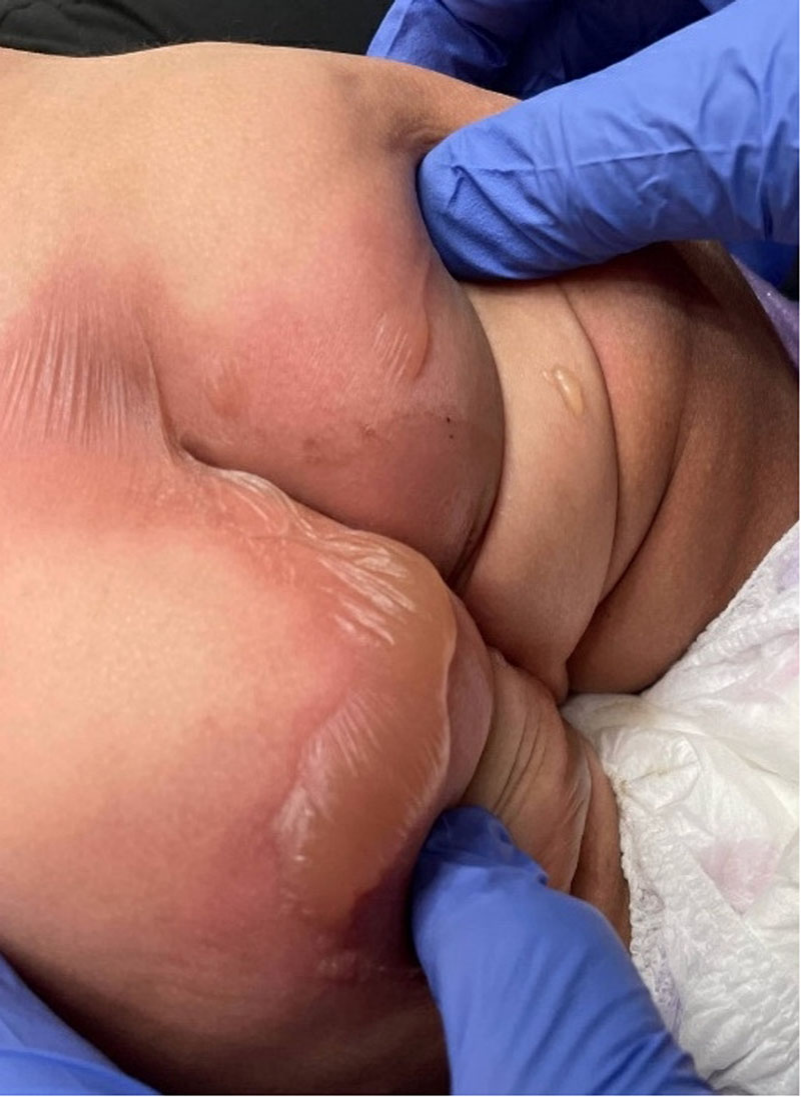
Sharply demarcated erythema in the gluteal region with three blisters: two small 1 cm bullae on the right gluteal area and one large, ~2.5% BSA, clear fluid‐filled roofed bulla measuring 4–5 cm on the medial left gluteal region, with sparing of the inguinal cleft and folds. BSA, body surface area.

A 2‐year‐old girl, previously healthy and not toilet‐trained (weight 12.5 kg), was brought to the emergency department after daycare staff observed erythema and blistering on her buttocks during a diaper change. The previous evening, around 11:00 p.m., the child accessed the refrigerator at home, and her mother witnessed her eat a square of chocolate‐flavored laxative chew that contained 15 mg of sennosides, approximately 1.2 mg/kg. She had no other medication or corrosives accessible, and no other toxic ingestions were reported or seen. Two loose stools occurred in the morning, with no perianal changes noted at that time. When she arrived at the ED, approximately 15 h after ingestion, her temperature was 37.2°C, and her other vital signs were normal. Physical examination revealed widespread erythema in the gluteal area, with three blisters: two small 1 cm bullae on the right buttock and one large, tense, erythematous, fluid‐filled bulla measuring 4–5 cm on the medial left buttock, sharply demarcated at the diaper margins and sparing the inguinal cleft and folds (Figure 1). No bruises, splash marks, or other injuries were seen. Systemic examination was unremarkable. The blisters were carefully unroofed, irrigated with sterile saline, covered with mupirocin 2% ointment, and petrolatum‐impregnated gauze. She was prescribed oral Cephalexin (50 mg/kg/day TID) for 5 days. Social work and Child Protective Services were consulted due to concerns about possible non‐accidental trauma; however, the witnessed ingestion and injury pattern supported an accidental chemical burn. A follow‐up 2 weeks later showed complete healing with no pigmentary changes or scarring.

## 3 DISCUSSION

Our case highlights the dermatological complications associated with sennoside ingestion in children. Sennoside is commonly used as a laxative; its ingestion, especially in young children, can lead to various adverse effects beyond gastrointestinal symptoms.^1^ Sennosides are the active components from plants in the genus Senna, which are derivatives of anthraquinone glycosides. Bacterial enzymes in the intestines break down sennosides, releasing the active compound, rhein‐9‐anthrone, which produces a laxative effect. The glycosides of rhein dianthrone are inactive and water‐soluble, preventing their passage through the lipophilic membranes of the small intestine.^6^ The exact mechanism of blistering remains unclear; it is hypothesized that bacterial metabolism of sennosides to rhein anthrone causes a caustic effect on the skin, particularly under occlusive conditions, such as those in diapers.^2^ The minimum duration of contact needed to cause injury is not well defined; reported cases occurred after overnight or prolonged exposure in diapered children.^7^

This case brings to the forefront the importance of thorough examination and accurate history‐taking. It also emphasizes considering laxative‐induced dermatitis as a differential diagnosis in patients with diaper‐area burns. Previous literature has documented blistering dermatitis following ingestion or administration of sennosides in young children aged 23–42 months, particularly when there is prolonged contact with fecal matter during the night, or extensive diarrhea with a long period of stool‐to‐skin contact.^3^, ^4^ Our patient's pattern of injury—erythema and blistering sparing skin folds—aligns with previously reported presentations and may mimic signs of non‐accidental trauma.^3^ This resemblance underscores the critical need for a detailed clinical history to prevent unnecessary trauma to the family from false abuse accusations.^8^

Our findings are similar to those reported by Schweitz et al.,^7^ who described a toddler developing bullous dermatitis localized to the diaper area after ingesting chocolate senna. As in our non‐toilet‐trained patient, the lesions were distributed to areas in contact with stool in occlusion.^7^ In our case, the ingested dose was smaller (15 mg vs. 45–60 mg), and the time to lesion was longer. However, they shared certain features: age, ingestion of a flavored sennoside, acute onset, localization in the diaper area with sparing of the gluteal folds and creases, and bullous morphology.^7^ This distinct blistering was also reported in children treated with senna for chronic constipation, emphasizing the importance of recognizing senna‐induced contact dermatitis as a distinct clinical entity.^4^, ^9^ This reinforces clinical awareness and the avoidance of unnecessary investigations in the absence of other risk factors and systemic signs.

Reported cases show that blistering episodes associated with both accidental ingestion and long‐term treatment with senna involved high doses; this is similar to our case, where the ingested dose was 1.2 mg/kg, three times higher than the standard dosing for this age group: 2.5–5 mg/day for ages 2–6 years.^10^ While most literature reports, like ours, involve accidental ingestion, other case reports, such as Cogley et al., remind us that therapeutic sennoside doses also pose risks, especially in infants and toddlers. In these cases, contact burn injuries occurred overnight in all diaper‐wearing patients, suggesting prolonged stool‐skin contact times. This is similar to our case, which also involved prolonged stool‐skin contact.^4^, ^9^

## 4 CONCLUSION

Laxative‐induced contact burns resulting from sennoside ingestion represent a rare yet significant clinical issue, especially in children. This case underscores the importance of recognizing potential dermatological complications in children with a history of sennoside ingestion to prevent the misdiagnosis of child abuse. It also highlights the necessity of adhering to administration protocols for sennosides, specifically administering the medication during daytime hours to avoid skin contact overnight. Furthermore, in cases of accidental ingestion, it is recommended that parents or caregivers increase the frequency of diaper changes to reduce stool‐to‐skin contact and apply occlusive barriers, such as petrolatum jelly, until diarrhea resolves. Additionally, families with other children or relatives who also use sennosides should receive appropriate counseling. The use of child‐resistant packaging and secure storage solutions can further reduce the risk of accidental ingestion of sennoside products.

## CONFLICT OF INTEREST STATEMENT

Drs. Tolulope Olorunsogo and Ahmad R. Miri are current members of NASPGHAN and are in good standing. The remaining author declares no conflicts of interest.

## ETHICS STATEMENT

The authors confirm that verbal informed consent for publication has been obtained from the patient's legal guardian. Drs. Tolulope Olorunsogo, Christian O. Oarhe, and Ahmad R. Miri participated in drafting this case report, and all approved the final version. Dr. Christian O. Oarhe obtained consent for publication from the patient's legal guardian.
